# The High-Energy Ion Telescope (HIT) for the Interstellar Mapping And Acceleration Probe (IMAP) Mission

**DOI:** 10.1007/s11214-026-01279-6

**Published:** 2026-02-27

**Authors:** E. R. Christian, J. G. Mitchell, A. Bruno, D. Albaijes, S. Bailey Newman, R. C. Boggs, M. K. Choi, C. M. S. Cohen, W. R. Cook, Y. S. Darwish, A. J. Davis, G. A. de Nolfo, J. J. Dumonthier, R. D. Garnett, M. Gkioulidou, K. J. Gregory, D. Guzman Garcia, J. P. Haas, C. Hollenhorst, M. Jeunon, M. A. Kaiser, S. G. Kanekal, B. Kecman, R. A. Leske, J. D. Letzer, I. Liceaga Indart, D. J. McComas, B. McNeel, G. D. Muro, J. T. Nolan, M. Ogindo, P. Petrowich, W. J. Reaves, T. P. Rosnack, N. Saghafi, C. L. Salerno, N. A. Schwadron, M. A. Silber, E. S. Smith, M. T. Smith, G. Suárez, T. Tatoli, C. P. Tiu, M. E. Wiedenbeck, M. H. Windhausen, Z. Xu

**Affiliations:** 1 Goddard Space Flight Center, Greenbelt, MD 20771 USA; 2 The Catholic University of America, Washington, DC 20064 USA; 3 California Institute of Technology, Pasadena, CA 91125 USA; 4 Kennedy Space Center, Merritt Island, FL 32899 USA; 5 Johns Hopkins University, Applied Physics Laboratory, Laurel, MD 20723 USA; 6 Aerodyne, Cape Canaveral, FL 32920 USA; 7 Department of Astrophysical Sciences, Princeton University, Princeton, NJ 08544 USA; 8 Nolan Engineering LLC, Greenbelt, MD 20771 USA; 9 Axient LLC, Columbia, MD 21045 USA; 10 Science Systems and Applications (SSAI), Lanham, MD 20706 USA; 11 Peraton, Greenbelt, MD 20770 USA; 12 Space Science Center, University of New Hampshire, Durham, NH 03824 USA; 13 KBR Wyle, Lexington Park, MD 20653 USA; 14 JPL, Pasadena, CA 91011 USA; 15 Columbus Technologies and Services, Inc., Greenbelt, MD 20770 USA

## Abstract

The Interstellar Mapping and Acceleration Probe (IMAP; McComas et al. in Space Sci Rev 214:116, 2018a; McComas et al. in Space Sci Rev 221:100, 2025) is a NASA mission designed to study two of the most important issues in heliophysics: the interaction of the solar wind with the local interstellar medium and the acceleration of energetic particles. These two puzzles are surprisingly intertwined because particle acceleration in the inner heliosphere plays a large role in the interactions at the edge of the heliosphere. The ten instruments on IMAP, situated near the Earth-Sun L1 Lagrange point, include both in situ and remote-sensing observations and are designed to work together to explore these questions. The High-energy Ion Telescope (HIT), described herein, measures in situ ions from hydrogen through nickel at energies ranging from a few MeV/nucleon to some tens of MeV/nucleon, depending upon species. While the primary focus of HIT is Solar Energetic Particles (SEPs), HIT is also sensitive to Anomalous Cosmic Rays (ACRs) and Galactic Cosmic Rays (GCRs) that are also present at these energies. Working with the other IMAP instruments, HIT studies how the variation in SEP intensities, spectra, anisotropies, and composition inform the acceleration processes that generate these particles, and, in turn, how these high-energy particles affect the outer heliosphere. This paper details the design and operation of the HIT instrument.

## Introduction

The High-energy Ion Telescope (HIT) is an energetic particle spectrometer that measures the highest energy in situ ions of the ten instruments on the NASA Interstellar Mapping and Acceleration Probe (IMAP; McComas et al. 2018a, 2025). HIT uses multiple layers of silicon solid-state detectors (SSDs) to implement the classic $dE$/$dx$ vs. E_total_ method (e.g. Stone et al. 1998) that determines the electric charge and energy of incoming energetic ions with incident energies of a few MeV/nucleon to some tens of MeV/nucleon. At these energies, the ions are fully stripped as they transit through the SSDs, so this corresponds to a measurement of the elemental species. When plotting $dE$/$dx$ vs. E_total_, different species are separated based on their electric charge (Z) and mass (M). The HIT resolution can cleanly identify all the common elements up through nickel. It is also sufficient to separate ^3^He from the more abundant ^4^He. This is a key instrumental capability as ^3^He is an important tracer of particle acceleration. HIT has considerable heritage from the Solar Terrestrial Relations Observatory (STEREO; Kaiser et al. 2008) Low-Energy Telescope (LET; Mewaldt et al. 2008).

HIT has ten apertures, two of which (sunward and anti-sunward) have been modified from the heritage STEREO/LET design to be sensitive to electrons from a few hundred keV to a few MeV. These electron apertures are specifically for space weather science as part of the IMAP Active Link for Real-Time system (I-ALiRT; Lee et al. 2025). They help further the understanding of SEP events by using high-energy electron intensities to better predict the high-energy proton intensities which follow (Posner 2007). The other eight apertures are focused on energetic ions and oriented so that they scan the sky as IMAP spins at 4 rpm, giving a 4$\pi $ field of view. The processed data mostly consists of full-sky-averaged intensities for a wide range of elements and energies, but there are also intensities for ten common species/energies that are separated into 120 22.5° x 24° bins for anisotropy observations.

## Science Requirements

HIT measures the highest-energy ions on IMAP and is responsible for observing accelerated solar energetic particles (SEPs), anomalous cosmic rays (ACRs) and low-energy galactic cosmic rays (GCRs) (Cohen et al. 2026). SEPs are accelerated in solar eruptive events, including coronal mass ejection (CME)-driven interplanetary shocks and magnetic reconnection in the form of solar flares and jets (e.g., Reames 2013; Desai and Giacalone 2016). In these events, charged particles can be accelerated to ∼GeV energies. Despite decades of study, the physical mechanisms that accelerate particles up to these energies are still not fully understood. However, a variety of SEP event characteristics, as well as contextual information (e.g., associated radio bursts, magnetic field signatures, etc.), can be utilized to better understand the acceleration, and transport, of these particles. In particular, the event duration, elemental/isotopic (e.g., ^3^He/^4^He) composition, anisotropy with respect to the local magnetic field, spectral features, and time series characteristics can provide important clues to determine the acceleration and transport mechanisms that affect these particle populations and produce the observed characteristics. HIT data provide measurements of all the above-mentioned SEP event characteristics to help improve our understanding of these features and how they relate to the processes that accelerate these particles.

As they propagate through the interplanetary medium, energetic particles are affected by a variety of processes including solar-wind turbulence, field-line random walk, and interaction with solar-wind structures. These all contribute to producing the time profile, anisotropy, and spectral features of the event, making it challenging to identify whether certain observed features arise from the acceleration mechanism or are modified by transport processes. Understanding the evolution of features as the SEP event propagates away from the Sun can deconvolve these effects. IMAP provides unique insights into particle acceleration and transport by comparing the observed features with missions in the very inner heliosphere such as Parker Solar Probe (Fox et al. 2016) and Solar Orbiter (Müller et al. 2020) and other spacecraft at L1. In particular, HIT provides unique anisotropy data of the full 4$\pi $ sr sky, which, when combined with local magnetic field measurements, improving our understanding of SEP transport.

In addition to the physical acceleration and transport mechanisms, the source of particles available to be energized by these processes is under debate. Whether particles can be energized directly out of the solar wind or if a suprathermal “seed” population is required remains unclear (e.g., Desai et al. 2006). With its ability to resolve particle species from H to Ni, and ^3^He from ^4^He isotopes, HIT provides key measurements to better understand SEP acceleration and seed populations. With combined measurements from HIT, the Compact Dual Ion Composition Experiment (CoDICE; Livi et al. 2026), the Solar Wind and Pickup Ion instrument (SWAPI; Rankin et al. 2025), and the Solar Wind Electron instrument (SWE; Skoug et al. 2026) the IMAP in-situ particle instruments will continue the study of this highly debated aspect of particle acceleration.

In addition to solar eruptive events, charged particles can be accelerated by solar wind stream interaction regions (SIRs; see e.g. Richardson 2018 and references therein). SIRs are formed when a fast solar-wind stream overtakes a slow solar-wind stream resulting in a significant pressure gradient at the stream interface. Depending on the relative speeds of these streams, compression regions or interplanetary shocks (a forward and reverse shock pair) can form and accelerate particles (e.g., Fisk and Lee 1980; Gosling and Pizzo 1999). In-situ measurements of SIRs and their resultant energetic particles by HIT provide key contextual data to understand variations in the energetic neutral atoms (ENAs) and ACRs propagating from the outer heliosphere (McComas et al. 2018b). It has been shown that pressure pulses produced by SIRs and fast CMEs can play a significant role in modifying conditions in the outer heliosphere. These measurements are critical to connecting inner heliospheric structures with effects observed in the outer heliosphere.

HIT also measures ACRs and low-energy GCRs. ACRs are believed to be produced by the acceleration of pickup ions at the blunt solar wind termination shock (McComas and Schwadron 2006; Giacalone et al. 2022), however, the exact acceleration mechanism is still not fully understood. Similar to the ENA observations made by other IMAP instruments, measurements of ACRs by HIT are able to probe their acceleration at the termination shock to better understand how variations in the heliospheric plasma environment, including solar-cycle variations, affect these populations. As well, HIT measurements of GCRs are used to study the effect of solar modulation on these populations and provide contextual data to outer heliospheric measurements performed by IMAP.

Due to their episodic nature, SEPs are highly variable in intensity, composition, and arrival direction, whereas the ACRs and GCRs vary more slowly due to transient strong magnetic structures and the solar cycle modulation effects. The HIT Science Traceability Matrix is shown in Table 1. HIT’s focus is on IMAP’s fourth science goal (McComas et al. 2025). The calibration and testing performed on the HIT flight model (FM) show that HIT meets or exceeds all the instrument requirements.

**Table 1:**
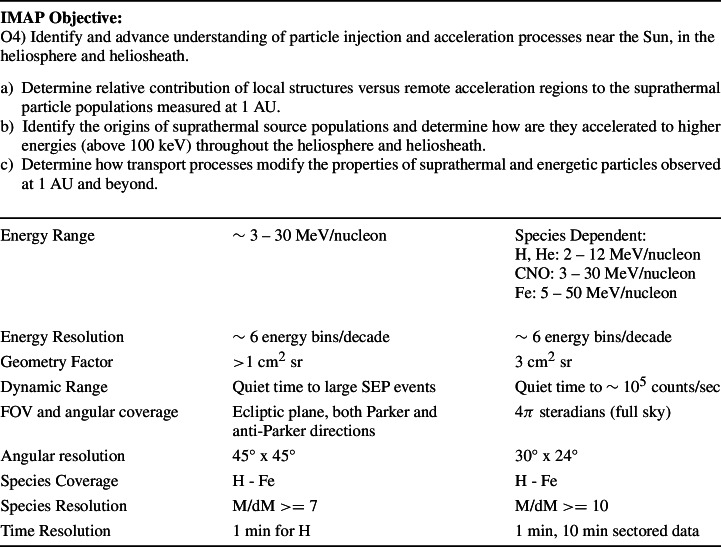
HIT Science Traceability Matrix

The science background for the HIT observations is described in detail in Cohen et al. (2026).

## HIT Sensor

### Overview

**Fig. 1:**
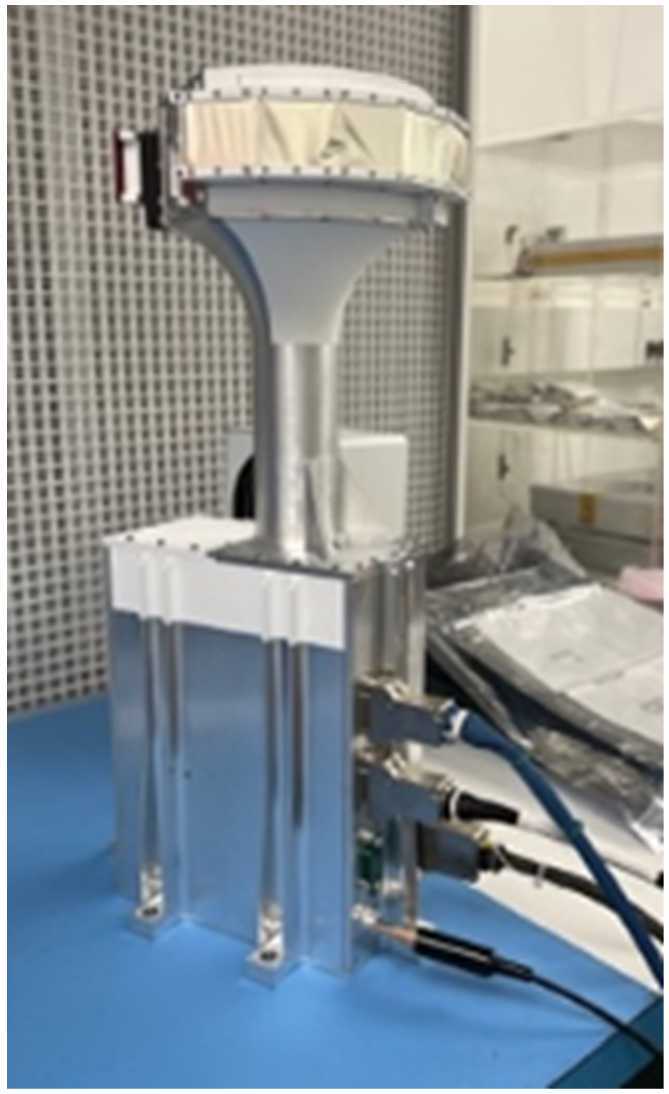
(Photograph of the HIT Flight Model undergoing bench testing

**Fig. 2:**
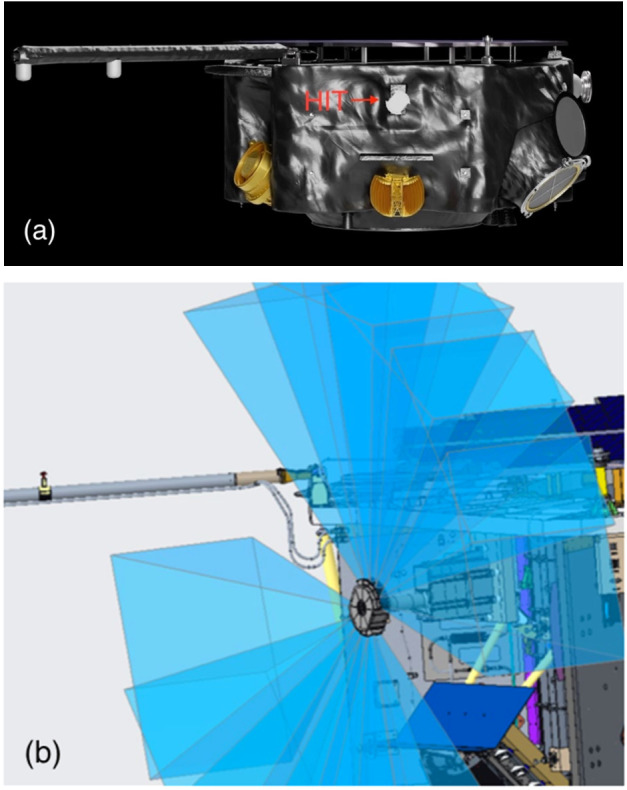
(a) Position of the HIT instrument on the IMAP spacecraft. (b) HIT FOV

Figure 1 shows the fully-assembled Flight Model (FM) of the HIT instrument. It is approximately 46x23x18 cm (18x9x7 inches) and consists of three components: an electronics box that is mounted to the spacecraft; a sensor head that contains the detectors and the front-end electronics (FEE); and a bracket that holds the sensor head so that it has a clear field-of-view (FOV). The ten viewing sectors are arranged in two fans that are perpendicular to the radial vector, which is perpendicular to the spacecraft spin axis (see Fig. 2). That radial vector is parallel to the axis along the cylindrical bracket. The eight sectors focused on ion measurements sweep out the entire 4$\pi $ sr sky as the spacecraft spins. The remaining two electron sectors sweep out cones in the sunward and anti-sunward directions.

**Fig. 3:**
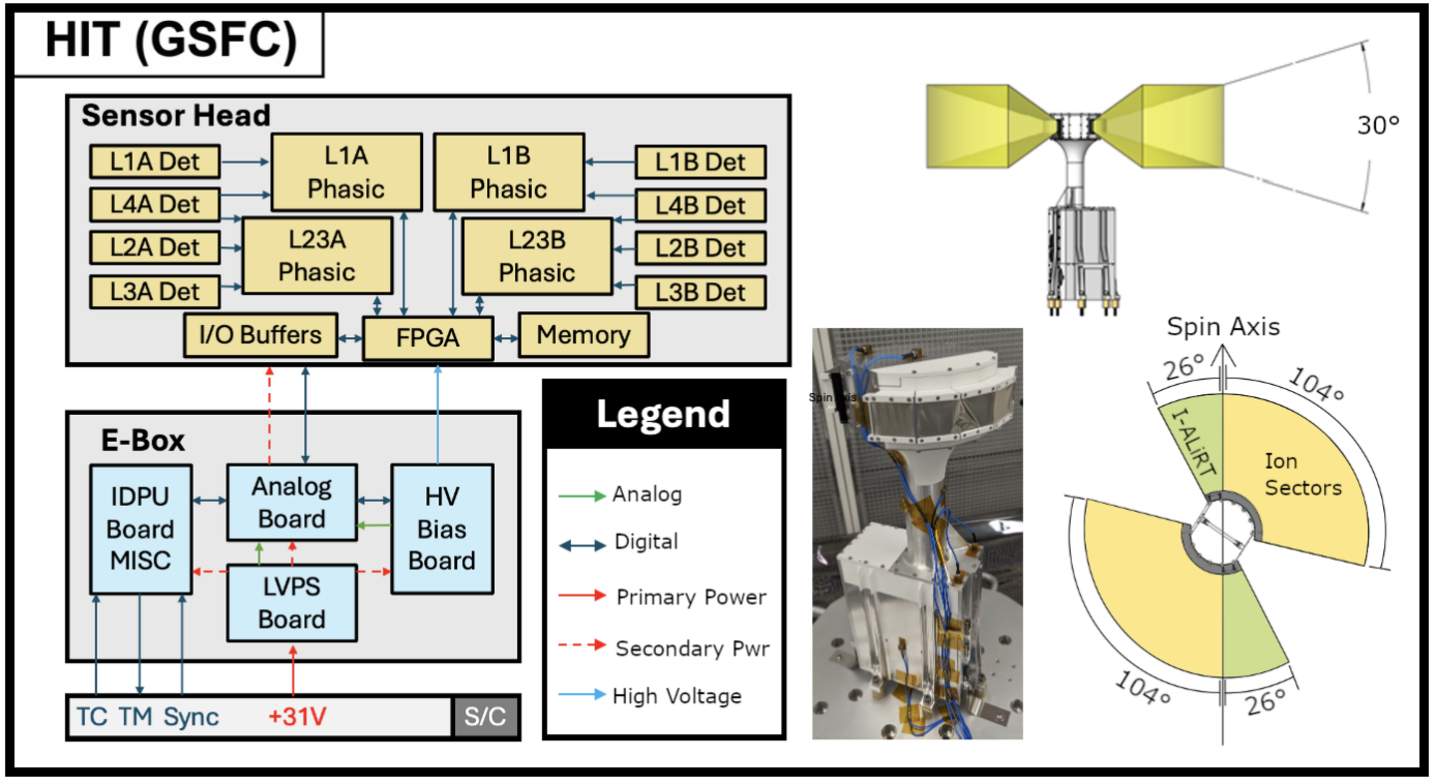
Summary of the key elements of the HIT instrument, with a block diagram, photo of the flight instrument being prepared for vibration testing, and the FOVs

A high-level block diagram of HIT (Fig. 3) includes four electronics boards in the electronics box (blue), and the components in the sensor head (orange). The FOV is shown in Fig. 3 for the two I-ALiRT sectors (green) and the two fans of four each ion sectors (orange).

#### Parameters and Resources

HIT is a compact, lightweight instrument with a wide FOV and the unique ability to measure energetic particle anisotropy for the entire 4$\pi $ sr sky. However, tradeoffs had to be made to fit within the alloted resources. HIT deliberately does not measure higher energy SEPs, and the resolution and geometry factor allow HIT to measure all the most common elements up to nickel without isotopic resolution except for ^3^He and ^4^He. The important parameters for the overall instrument are shown in Table 2.

**Table 2:** Summary of HIT Parameters and Resources

Parameter	Performance
Instrument Type	Silicon SSD ΔE/E_tot_ ion and electron spectrometer
Species	e^−^, H, ^3^He, ^4^He, C, N, O, Ne, Na, Mg, Al, Si, S, Ar, Ca, Fe, and Ni
Energy Range	∼1 MeV/nucleon to ∼70 MeV/nucleon, species dependent
Energy Resolution	∼10%
Time Resolution	1 Minute
Ion FOV	Instantaneous, two fans, 108° x 30°, w/spin 4*π* steradians (sr)
Ion AΩ	3 cm^2^ sr
Electron FOV	∼30° half-angle cone, sunward and anti-sunward
Mass	3.50 kg
Power	5.96 W
Telemetry	745 bps

### Silicon Detectors

**Fig. 4:**
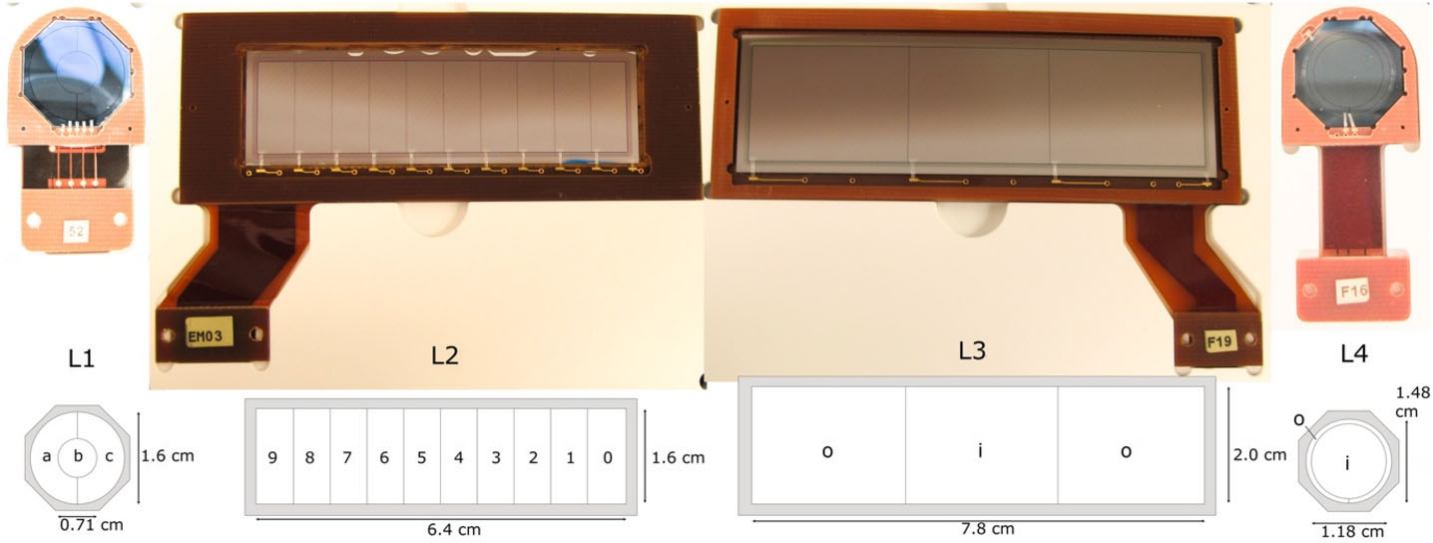
Photos and diagrams of the four HIT detector types. The ten L1 detectors are segmented into three active regions (a, b, c), the two L2 detectors have ten segments (0 – 9), the L3s have three regions (o, i, o) with the outer two (o) electrically tied together. The L4 detectors have an outer annulus (o) and an inner bullseye (i)

The core of HIT is the SSDs. There are 16 detectors of four different designs. All the SSDs were manufactured by Micron Semiconductor LTD. Figure 4 shows photographs and designs for the different styles and Table 3 lists the individual detector thicknesses.

**Table 3:**
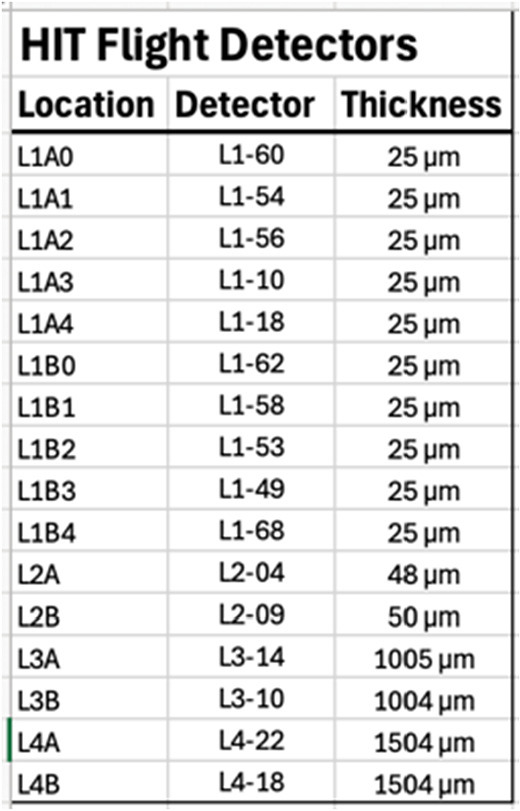
Flight Detectors and Thicknesses

**Fig. 5:**
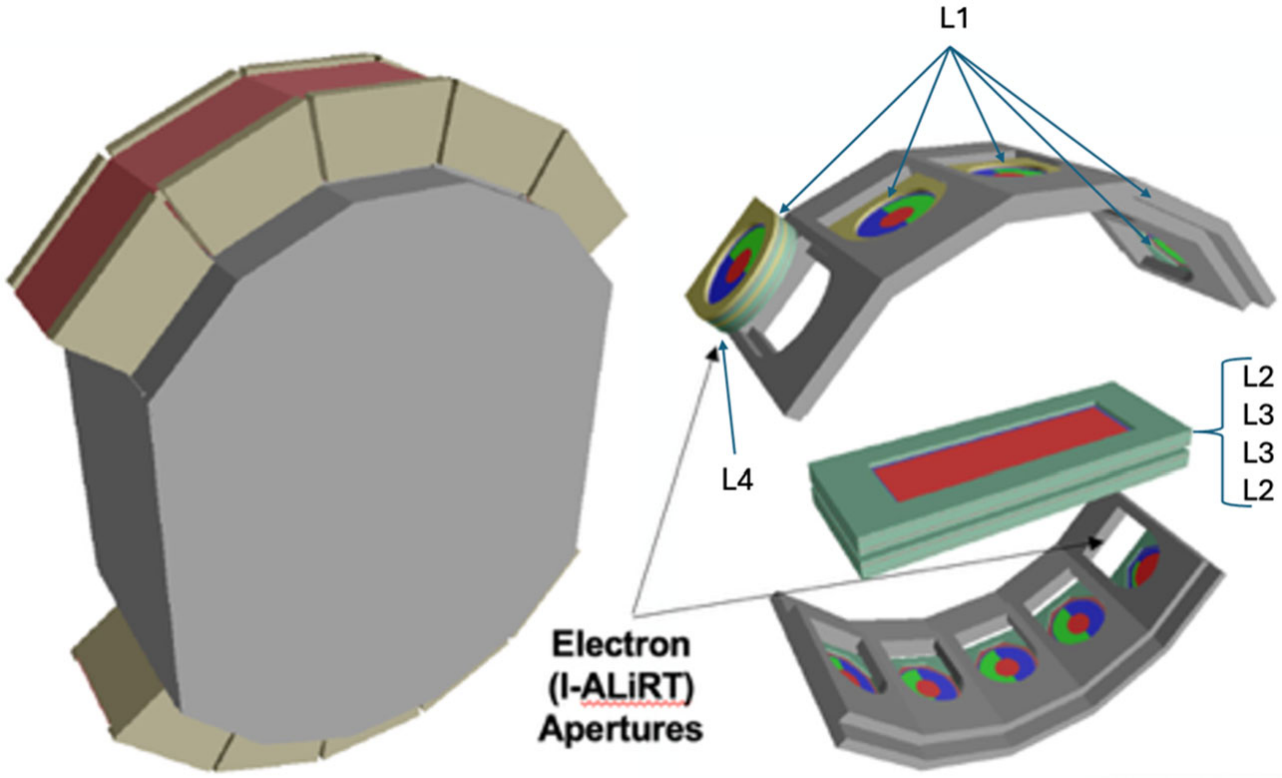
Sensor head exterior (left) and corresponding location of the sixteen silicon SSDs (right)

As shown in Fig. 5, the ten 25 $\mu $m thick L1 detectors are located at the front of each aperture, protected from dust and UV by two Kapton windows, each 8 $\mu $m thick. The front window has a thermal coating on the front and aluminum on the rear. The back window is aluminized on both sides. At the center of the sensor head is a stack of four detectors, two 50 $\mu $m L2s on the outside and two 1000 $\mu $m L3s in the middle (Fig. 5). For two of the ten apertures, the L1 detectors are moved outward to leave room for two 1500 $\mu $m L4 detectors directly behind. These are the I-ALiRT apertures that are sensitive to electrons (see Sect. 4.2 below).

The photos in Fig. 4 show the segmented junction side of all the detectors. The opposite (ohmic) surface of all detectors consists of a single contact covering the entire area. Detectors are installed by the manufacturer in multilayer G10 circuit boards with Kapton/copper flexible leads and connected with triply redundant 25 $\mu $m wire bonds. The segmentation in the L1 and L2 detectors is used onboard to determine trajectory and make pathlength corrections to the measured energy loss, which improves the particle identification (see Sect. 4.4).

### Front-End Electronics

The top disc of HIT (the sensor head) contains the silicon SSDs and the analog electronics required to analyze them, the Front-End Electronics (FEE). The FEE is a rigid-flex printed circuit board (PCB) assembly that wraps above and below the detectors, with two rigid sections connected by a flexible section. There is also a long flexible section that extends down the neck of the HIT chassis to connect to the HIT electronics box. The FEE PCB was manufactured as one single printed circuit board assembly.

#### Pulse Height Analysis Integrated Circuits (PHASICs)

As each charged particle passes through a HIT SSD, it creates an ionization track with the total ionization proportional to the energy lost by the particle in that detector. That ionization charge is digitized in custom Pulse Height Analysis System Integrated Circuits (PHASICs). The PHASICs were designed for the In situ Measurements of Particles And CME Transients (IMPACT; Luhmann et al. 2008) investigation on the STEREO mission (Kaiser et al. 2008) and subsequently radiation-hardened for the Integrated Science Investigation of the Sun (IS⊙IS) instrument suite (McComas et al. 2016) on the Parker Solar Probe mission (Fox et al. 2016). They are radiation-hardened versions (100 krad) based on similar designs that have flown in space for more than fifty years (Marshall and Halpern 1968). HIT uses four PHASICs, each of which has 16 dual-gain pulse height analyzer (PHA) channels. Each channel has a charge-sensitive preamplifier that drives low-gain and high-gain post-amplifiers independently. This results in a large dynamic range, needed to analyze electrons through nickel. The PHASICs programmable thresholds are used to internally generate counts (number of triggers) for each channel/gain combination. The PHASICs also contain several programmable OR-gates that are used to create the trigger of good events (Sect. 3.3.3) and internal precision test pulsers that provide functional testing and calibration of the circuit.

#### FEE Field-Programmable Gate Array

The FEE includes an RTAX2000S Field-Programmable Gate Array (FPGA) that is used to control and readout the PHASICs. The PHASICs are read out using a 24-bit tri-state bus using a token ring architecture and a sparse readout. The FPGA collects housekeeping data and configures test reference voltages for the PHASICs. It also includes two communication channels to the Instrument Data Processing Unit (IDPU), a transmission (unidirectional) science channel at 1 Mbaud LVDS (low-voltage differential signaling) and a command/housekeeping bi-directional channel (3.3 V TTL at 115,200 baud). It is configured as a state machine with no external programming.

#### Trigger

**Fig. 6:**
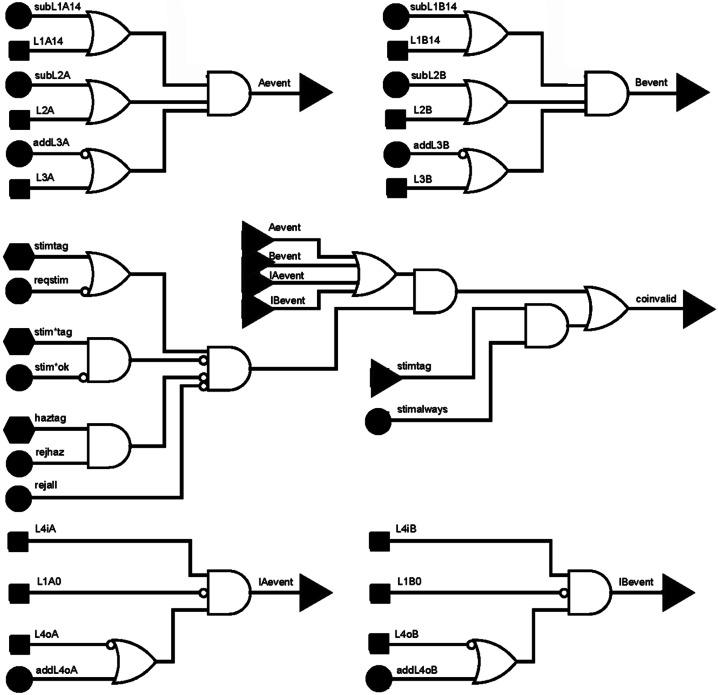
Logic tree of fast (hardware) trigger. Input signals that are black squares are the programmable or-gates from the PHASICs, black circles are memory bits that allow the trigger to be programmed in flight, hexagons are signals derived in the event, and the black triangles are connected elsewhere

Each of the sixteen channels on each of the four PHASICs has two (high-gain and low-gain) programmable discriminators. Any combination of those thirty-two signals can be logically OR’d together into three trigger signals (per PHASIC) that are labeled gor1, gor2, and gor3 (global or). These signals are described in Table 4 and feed into the fast trigger logic table shown in Fig. 6.

**Table 4:** Definition of global ‘or’ gates generated by the PHASICs

PHASIC	Signal	Label	Description
L1A	gor0	L1A14	All three segments (a, b, c) on each of the four non-I-ALiRT apertures, A-side
L1A	gor1	L1A0	All three segments (a, b, c) on the I-ALiRT aperture, A-side
L1A	gor2	L4oA	Outer ring (guard) of the L4 detector, A-side
L23A	gor0	L2A	All ten segments of the L2 detector, A-side
L23A	gor1	L3A	Both channels (three segments) of the L3 detector, A-side
L23A	gor2	L4iA	Inner active region, L4 detector, A-side
L1B	gor0	L1B14	All three segments (a, b, c) on each of the four non-I-ALiRT apertures, B-side
L1B	gor1	L1B0	All three segments (a, b, c) on the I-ALiRT aperture, B-side
L1B	gor2	L4oB	Outer ring (guard) of the L4 detector, B-side
L23B	gor0	L2B	All ten segments of the L2 detector, B-side
L23B	gor1	L3B	Both channels (three segments) of the L3 detector, B-side
L23B	gor2	L4iB	Inner active region, L4 detector, B-side

The instrument trigger is implemented solely in hardware and uses the signals from all channels that are higher than threshold as signified by the hardware gates (gors) from the PHASICs. If an event meets the trigger conditions shown in Fig. 6, the event gets stored in an output buffer to get sent to the IDPU. Only these events are processed and categorized by the flight software (FSW). If discriminators fire and the conditions for a valid event are not met, digitization will finish, but the values will be discarded.

The primary trigger for ions requires a signal in an L1 detector and the corresponding L2 (Fig. 6, top two subtrees). Input signals either come from the programmable or’s in the PHASICs (black squares) or memory bits (black circles) that allow in-flight adjustment of the trigger conditions. The primary trigger of I-ALiRT electrons is L4i without a signal in the corresponding L1 or L4o (Fig. 6, bottom two subtrees). There will be some background events from very high energy protons that are minimum ionizing when they pass through L1, and from high energy events that come through the instrument from the back side.

The middle subtree shows how the other types of events fit into the trigger. Stimtag are events generated by the onboard pulser and reqstim (require stim tag) cause only stim events to be transmitted. The stim*tag are stim events that incorrectly fire on the negative-going edge of the stim pulse and are usually ignored. The hazard flag (haztag) is true when two trigger events come too close together in time.

#### Thresholds

All of the channels have programmable discriminators with separate values for high- and low-gain. These are tuned by using the measured singles rates and are adjusted so that false (noise) triggers are at a low enough rate (less than a few tens per second) that accidental coincidences do not overwhelm the system. The most constrained thresholds are the ones for the L1A0 and L1B0 detectors (the I-ALiRT apertures) which are used as anti-coincidence to discriminate protons < 100 MeV and electrons <a few MeV. HIT launches with thresholds determined in the lab, but as part of commissioning, these thresholds are adjusted for the noise environment in flight. The design goals for the thresholds are 100 keV for the L1A0 and L1B0 detectors, 200 keV for the other L1 detectors, 300 keV for the L2 and L4 detectors, and 500 keV for the L3 detectors.

#### Dynamic Thresholds

During SEP events, the intensity of particles can increase by orders of magnitude, with the lower energy protons making up the vast majority of this population. This results in increased instrument deadtime and a greater likelihood of accidental coincidences between two different particles appearing to be a valid event. To overcome these challenges, HIT uses a “dynamic threshold” system to preserve the ability to measure heavy ions during large SEP events.

The dynamic threshold system autonomously monitors the summed count rates of certain detector segments. When the count rates surpass a set (commandable) threshold, the high-gain channels on certain detector segments are disabled, leaving only the low-gain channels and raising the effective measurement threshold. Each of the 3 dynamic threshold modes are “cumulative” in the sense that the high-gain channels that are disabled in a lower mode remains disabled at higher modes. Table 5 describes which high-gain channels are disabled at each dynamic threshold mode. As the energetic particle intensity decreases, the instrument returns to lower dynamic threshold modes. This transition typically happens when the count rate is roughly half the rate required to step up in dynamic threshold mode (also commandable).

**Table 5:** Definition of the four Dynamic Threshold states

Dynamic threshold mode	Segments with high-gains disabled
DT0	None
DT1	Outer regions of eight ion L1 (not I-ALiRT apertures)
DT2	Center segment of all L1 apart from two center apertures (L1A2 and L1B2)
DT3	All L2 segments apart from 2 center detectors (i.e., L2A4, L2A5, L2B4, L2B5)

### Electronics Box

The base of the HIT instrument is the electronics box with four PCBs: the Instrument Data Processing Unit, the High-Voltage (Detector Bias) Power Supply, the Low-Voltage Power Supply, and the Analog Board which operates as an interface between the other e-box boards and the FEE.

#### Instrument Data Processing Unit (IDPU)

**Fig. 7:**
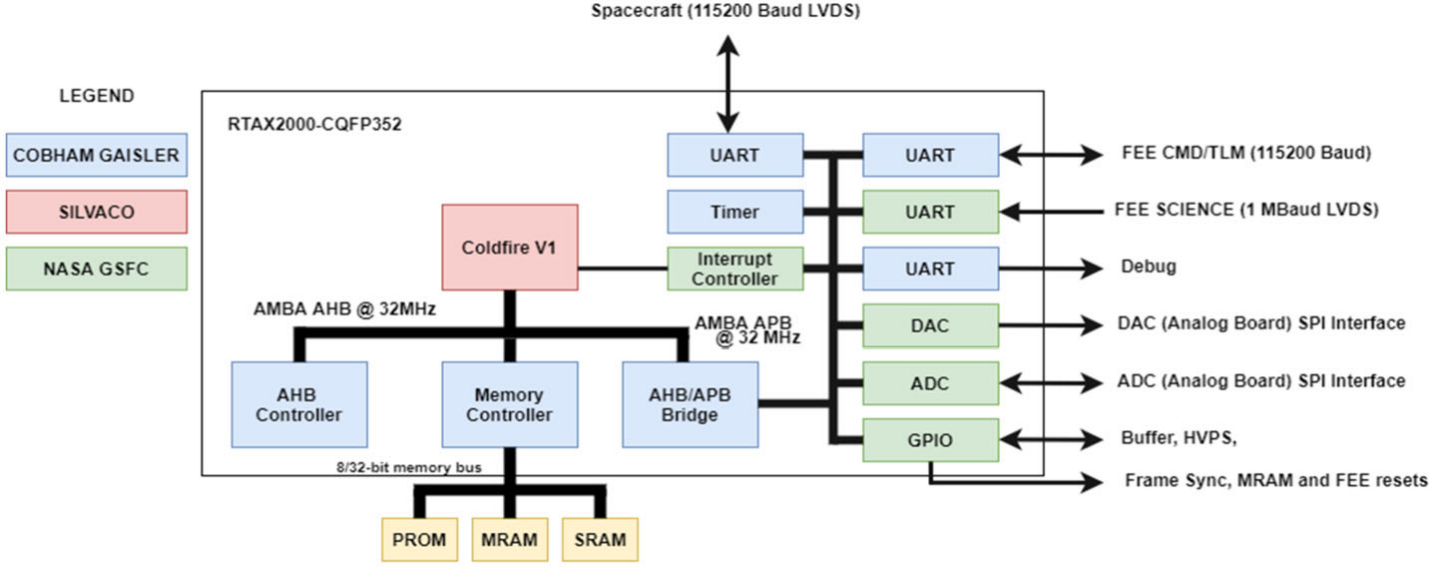
Block diagram of IDPU FPGA and interfaces

The IDPU is a printed circuit card assembly, which resides in the HIT EBOX. It consists of an RTAX2000S-CQ352 FPGA, which contains an embedded ColdFire V1 microprocessor system (see Fig. 7). Table 6 lists some of the key parameters for the IDPU.

**Table 6:** IDPU processor system parameters

Parameter	Performance
Primary Oscillator Frequency	16 MHz clock (32 MHz oscillator)
Boot Memory	256 Kb PROM
Volatile Storage	16 Mb SRAM
Non-Volatile Storage	16 Mb MRAM
Spacecraft Communication	115.2 kBaud UART over LVDS
FEE Commanding	115.2 kBaud UART over LVDS
Science Data Communication	1 Mbaud UART over LVDS

When the power is first supplied to the instrument, a Power On Reset (POR) circuit resets the FPGA on the IDPU. The boot loader code is then copied from PROM to SRAM. The boot loader sends a one-per-second autonomy packet to the spacecraft indicating aliveness but waits for ten seconds before starting the instrument software. If a command is received from the spacecraft during this ten-second wait, the instrument enters maintenance mode (MAINT) which allows new code and tables to be uploaded to either MRAM or SRAM. MAINT mode can only be exited by re-booting the instrument.

If no command is received during the 10-second wait, the flight software (FSW) starts. The MRAM holds multiple copies of the software (which can include different versions) and the copy used is selected at boot up. The selected version is copied into SRAM and begins to run. The instrument can also boot up directly with the FSW in SRAM, which allows a new software version to be uploaded in MAINT mode and run directly if there appears to be a problem with the MRAM.

#### Flight Software

The HIT FSW consists of two routines, a boot loader and a loadable application. The PROM-resident boot loader gains control of the IDPU on power-up or reset and then automatically loads a program image from MRAM if the integrity check passes or loads a secondary image if the primary image is corrupted. It can receive spacecraft commands and begins sending autonomy (“keep-alive”) packets once per second. The loader is capable of maintenance on MRAM tables and files or receiving and booting a program image directly from the spacecraft interface. It can receive and write a program image, file, or configuration table to MRAM or make a program image the primary or secondary boot image. It also can perform a complete MRAM file system check with option to correct errors and even reformat the MRAM if necessary. For safety, the MRAM is write-protected when the software is not actively writing tables or files. The design is inherited from a long line of instruments, with MRAM now replacing EEPROM.

Although the loadable application has heritage from the STEREO/LET software written in Forth, the software was completely converted to C on HIT. In addition, a number of changes have been made to the software, including a better separation of Look-up-Tables (LUT), a different spacecraft interface, spin sector rates, new I-ALiRT data, and new event types that involve L4. In addition, the STEREO/LET FEE FPGA included an embedded microprocessor whereas the HIT FEE is a purely state machine. This shifts some onboard analysis to the IDPU software.

##### Onboard Particle Identification

Onboard particle identification is done using a lookup table on the last two detectors the particle enters ($\Delta $E vs. E’). The energy loss in those two counters generate an index, in log space, into a 2D matrix calculated for the appropriate range. The value in the matrix is a particle ID that specifies both the species and the energy range.

##### Priority System

HIT also allocates about 70% of the telemetry to individual events, which are used to check the onboard particle identification. The events are transmitted with a priority system of 29 buffers that can hold up to eight events at a time. Events identified as belonging to an already full buffer are dropped after being counted. The events to be read out are selected by a “round-robin” system from a list of 240 entries. The “weight” assigned to a given buffer indicates the number of times the buffer appears on the list, so that buffers with heavier weights are read out more often. HIT only transmits approximately four events per second, so the “round-robin” takes about a minute when there are events in every buffer, which only occurs in a large SEP event. The weights were assigned according to the scientific priority of the class of events, as well as their expected frequency of occurrence.

##### Livetime Measurement

The fraction of time during which the HIT electronics are available to measure incident particles is referred to as the livetime. The instrument has small periods of deadtime due to the time it takes to record individual events. HIT utilizes an onboard pulser to measure the instrument livetime. Livetime STIM is pulsed in 9 out of every 10 seconds (in the last second, the ADC-Calibration STIM is pulsed). The Livetime STIM is pulsed 5 times per second in 54 seconds out of every minute, and thus if all Livetime STIM pulses are recorded, there would be 270 total Livetime STIM pulses per minute. The livetime counter records the number of Livetime STIM pulses it measures in each minute. The fraction of the livetime counter over 270 for each minute gives the overall livetime for that minute.

#### High Voltage Power Supply (HVPS)

The HVPS generates bias voltages to the sixteen silicon SSDs located in the sensor head. SSDs are operated as reverse-biased diodes and are fully functioning over a relatively broad range of bias in which the detectors are fully depleted but not suffering breakdown over-voltage. The board has three power supplies (PS) that power four output rails. One 250 V PS supplies 150 V – 250 V for the A-side L3 and L4 detectors, and one 250 V PS supplies 150 V – 250 V for the B-side L3 and L4 detectors. The third 25 V PS supplies both the 2 V – 20 V for the ten L1 detectors, and 2 V – 20 V for the two L2 detectors.

The leakage currents of the detectors are temperature dependent and also increase with time as the detectors undergo radiation damage. The nominal leakage current at operational temperature and beginning of life are a few $\mu $A or less per detector and the HVPSs are capable of supplying more than 0.5 mA each, which is sufficient headroom for decades of nominal operations.

#### Low Voltage Power Supply (LVPS)

The LVPS is the primary power interface between the spacecraft and the HIT instrument. On it, the IMAP bus voltage (+24 V to +35 V) is converted down to lower voltages through isolated topologies and fed to the Analog Board for distribution to the rest of the HIT instrument. Switching supplies generate four analog supply voltages (+12 V, −12 V, +5.7 V, and −5.7 V) and three digital supply voltages (+5.1 V, +3.4 V, and +2.0 V). There is built-in filtering of the input power and soft-start circuits are also included. The heater power (both survival and operational) are passive pass-throughs of the spacecraft bus voltage.

#### Analog Board

The Analog Board acts as a backplane to the HIT system, and all the inter-board communications, signals, and voltages pass through it. The DACs that control the detector high-voltage biases are on the Analog Board, as are the housekeeping ADCs. These ADCs are used to telemeter the bias voltages, board temperatures, the four analog supply voltages and the three digital supply voltages (see Sect. 3.4.4). The HIT survival heaters and thermostats are also on the Analog Board.

### Mechanical Design

**Fig. 8:**
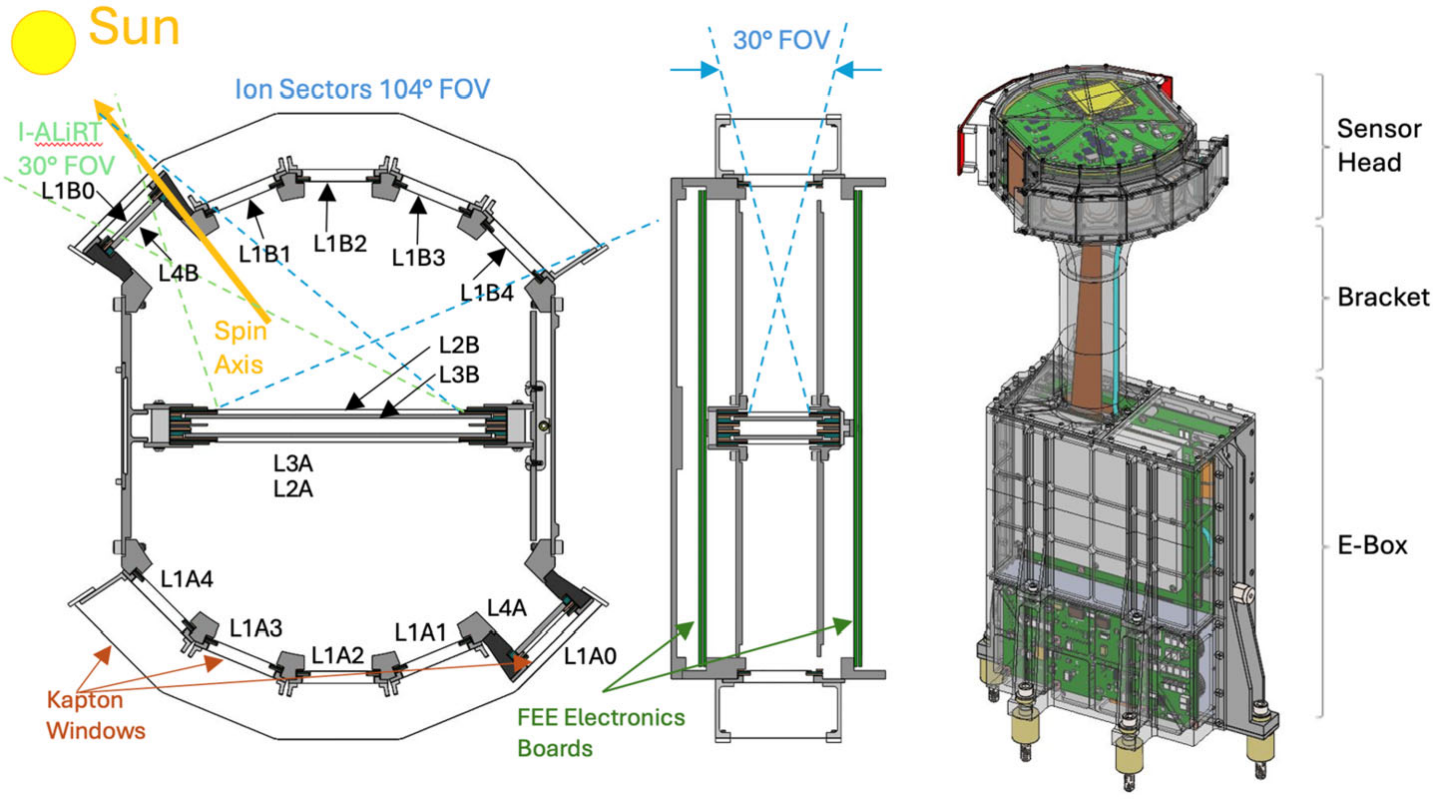
Simplified CAD drawings of the (left) HIT Sensor Head from the top, (center) HIT Sensor Head from the side, and (right) entire HIT instrument

The overall HIT mechanical design followed LET, an electronics box (E-box) as the base, a sensor head with the detectors and FEE, joined by a re-enforced cylindrical bracket (Fig. 8). The modifications were 1) addition of two ground straps (2.5 x 0.5 inches) made of 2 mil hardened pure aluminum foil attached from HIT chassis to the spacecraft structure, 2) a simple rotation of the sensor head, easily accommodated by the flexi-strip circuit that connects the sensor head to the Analog Board, and 3) the electronics were modified because a number of components, such as the FPGAs, were replaced with parts that are currently available, and the E-box electronics were simplified because the STEREO E-box supported more detectors than just the LET sensor head. The interior of the E-box was changed to match the four simplified electronics boards.

**Fig. 9:**
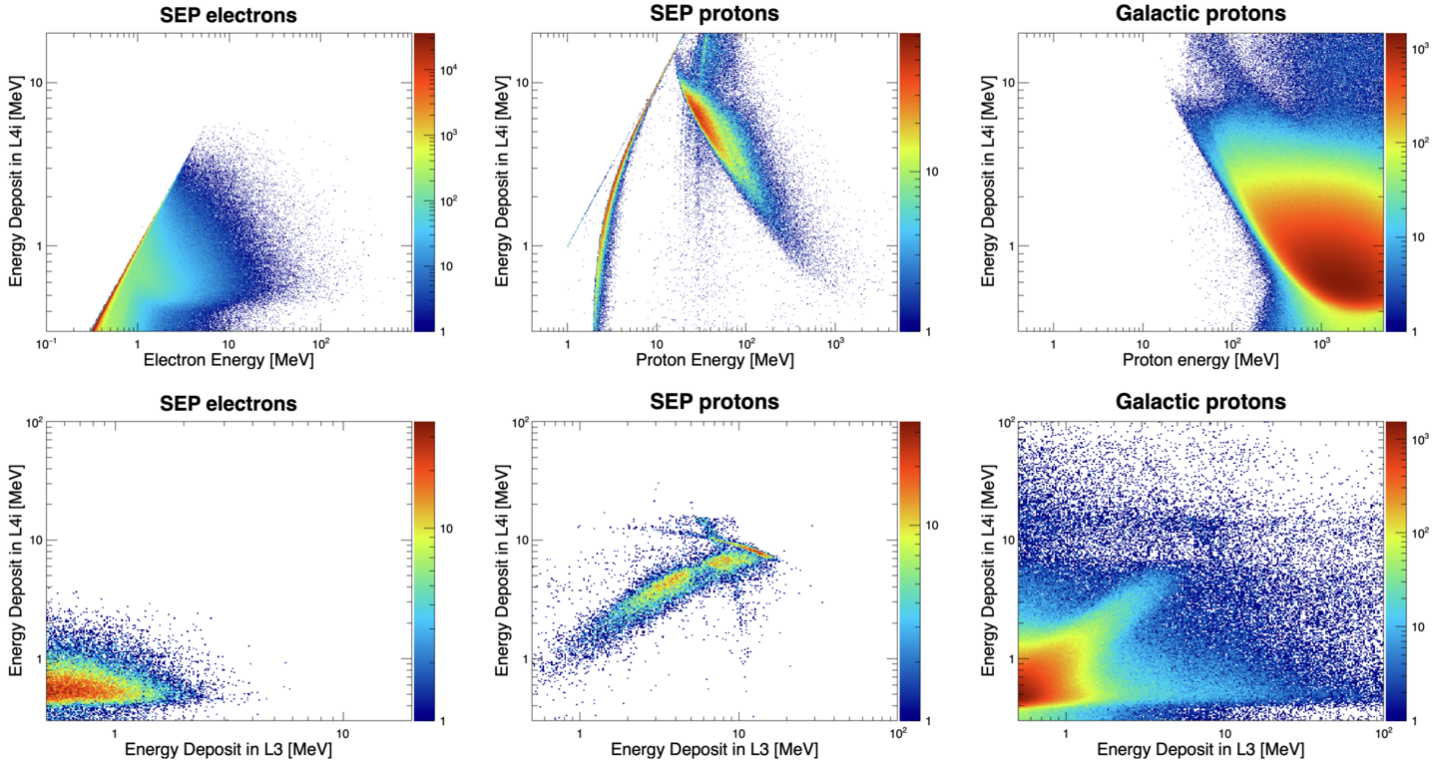
Top: distributions of the energy loss in L4i as a function of the incident energy. Results for SEP electrons and protons, and GCR protons are reported in the left, center and right panels, respectively. Bottom: corresponding L4i vs. L3 energy-loss distributions

Every attempt was made to keep the outer envelope of the instrument the same in order to minimize any secondary effects of the I-ALiRT modification. The L1A0 and L1B0 are identical to the other L1 detectors, but were moved outward to allow new L4 detectors to be placed directly behind them (see Fig. 9).

### Thermal Design

The HIT enclosure is thermally isolated from the spacecraft using Ultem 2300 bushings. The cold case occurs when the solar radiation is a minimum, the instruments are operating with the lowest heat dissipation in the electronic equipment, and the optical properties of the coatings have not been degraded. The hot case occurs when the solar radiation is at the maximum, the instruments are in operational mode with the highest heat dissipation, and the optical properties of the coatings are degraded. The HIT thermal design keeps HIT in the flight ranges of +25 °C to −20 °C for the electronics and +10 °C to −20 °C for the detectors, and +40 °C to −35 °C nonoperational. The cooling approach includes passive radiators, which radiate to deep space. The outer surfaces of the instrument that are neither radiator nor aperture windows are covered with a multilayer insulation that has a StaMet coated black Kapton outer layer. Two types of electrical heaters are employed: operational and survival heaters.

### Contamination Control

HIT follows all the standard NASA procedures to limit contamination to itself and the rest of the IMAP spacecraft and instruments. These include using material and processes with minimal outgassing properties, cleaning and baking all flight material, and using cleanroom or clean benches for assembly and testing. Because IMAP includes a sensitive magnetometer, HIT has been designed with minimal magnetic material and consideration of the magnetic effects of changing currents in the instrument. In addition, an external dry nitrogen purge is supplied to the instrument, which is directed into the sensor head near the SSDs.

## Sensor Performance Model

### GEANT4 Simulations

The HIT response was investigated with the Geant4 v11.3.2 package (Agostinelli et al. 2003). The simulation implements an accurate description of the electromagnetic and hadronic interactions undergone by incident particles (“FTFP BERT EMZ” physics list), as well as of the instrument geometry and materials based on mechanical drawings via the CAD interface CADMesh tool (Poole et al. 2012). The flux of the main 16 ion species measured by HIT (H, ^3^He, ^4^He, C, N, O, Ne, Na, Mg, Al, Si, S, Ar, Ca, Fe and Ni) was generated isotropically from a spherical surface surrounding the detector, with a power-law energy spectrum between 1 and 200 MeV/n with a −2.5 spectral index. The resulting count distributions were scaled accounting for the relative abundances based on Reames (2021). In particular, the intensity of ^3^He was set to be ∼10% of ^4^He.

### I-ALiRT

The two I-ALiRT apertures (A0 and B0) are equipped with an additional 1.6-cm-diameter detector, L4, located behind L1, aimed to measure relativistic electrons. The L4 design has been optimized by means of Monte-Carlo simulations to maximize the instrument response to electrons, while reducing as much as possible the dominant background associated with solar and galactic protons. After testing different thicknesses (1–1.5 mm) and configurations (single or double-layer sensor), a detector thickness of 1.5 mm was found to achieve the best performance. The 25-$\mu $m thick L1s are used as anti-coincidences, exploiting the fact that they are relatively insensitive to electrons, while they can be used to discard most protons (and ions); the hadron rejection is further improved by setting a lower threshold compared to the regular L1s. Since the basic trigger configuration of the I-ALiRT apertures only involves the L1 and L4 detectors, the corresponding field of view is significantly wider than the other L1 apertures, which require at least a hit in the corresponding L2. To reduce the acceptance, the L4 detector was segmented into a central circular sensor with a 1.3-cm diameter (denoted L4i), surrounded by an annular sensor (L4o) included as veto in the trigger logic. Consequently, electrons are selected as events producing a signal in L4i without a signal in the L1 and the L4o segments.

**Fig. 10:**
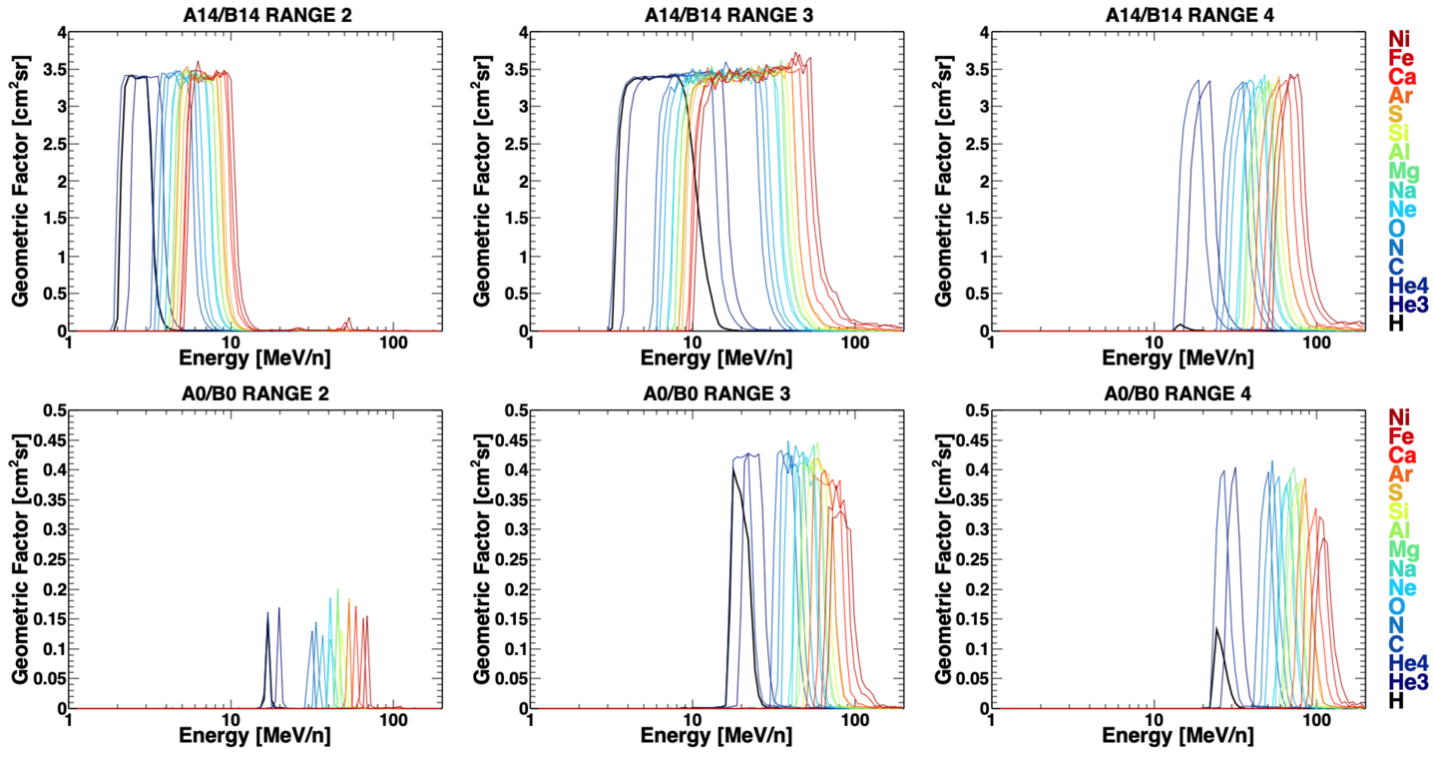
HIT geometry factors vs. energy/nucleon for Range 2 (left), Range 3 (center) and Range 4 (right). The top and bottom panels refer to the sum of A1–A4/B1–B4 apertures, and to the A0/B0 aperture, respectively. The ion species is indicated by the color code

The top panels in Fig. 9 demonstrate the L4i simulated response to electrons (left), SEP protons (center) and GCR protons (right), as a function of the incident energy. In the case of solar particles, a simple power-law spectrum with a −3 spectral index has been assumed; the number of events in each plot is arbitrary (i.e., no relative normalization). The minimum energy is determined by the ∼300 keV threshold of the L4 sensor. Due to the much shorter propagation time to 1 AU of electrons than protons of the same energy, the contamination in I-ALiRT from lower-energy protons is expected to be greatly reduced with respect to the data presented in the Fig. 10. In addition, highest energy electrons can reach the L3 detector, which can be used to support the hadron/electron discrimination, given the different L4i vs. L3 energy-loss distributions characterizing the three particle categories. Results are displayed in the bottom panels of Fig. 10.

### Geometry Factor

The factor of proportionality between the ion intensities and the measured count rates (corrected by the selection efficiency) is provided by the instrument collecting power. For an isotropic flux, the detector response can be characterized in terms of the geometric factor, which depends only on the telescope geometry and directional response. Along with the geometric dimensions and relative positions of the sensor elements, it accounts for the hadronic and electromagnetic interactions of incident particles with the instrument materials, including scattering and energy losses, e.g. associated with the emission of high energy electrons (important for heavy ions).

The HIT geometric factors of the different ion species were estimated with Monte-Carlo methods (e.g., Sullivan 1971). The calculation was performed for each range, accounting for the corresponding trigger requirements. Results are shown in Fig. 10, where the geometric factors are displayed as a function of kinetic energy per nucleon, with the color code indicating the ion species. The solid curves refer to the sum of the four detector apertures not associated with I-ALiRT (A1–A4 or B1–B4), peaking at ∼3.4 cm^2^sr. For comparison, the bottom panels display the results for the remaining aperture (A0 or B0), peaking at ∼0.15 cm^2^sr for Range2, and ∼0.4 cm^2^sr for Range3 and Range4, and characterized by higher thresholds, due to the energy loss in the L4 sensors.

### Cosine Correction

As discussed above, particle identification with HIT employs the dE/dx vs. total energy technique to determine the nuclear charge of detected ions. This approach relies on the analysis of the response tracks given by the distributions of the energy-loss signal from a detector that the particle fully penetrates ($\Delta $E) vs. the energy deposited in a following detector in which the particle is absorbed (E’). The tracks are broadened by the finite resolution of the instrument due to several effects, including the statistical nature of the ionization process. In addition, the energy-loss signal from a particle depends on the angle of incidence with respect to the detector, with a larger inclination corresponding to a longer path through the sensor material due to the oblique trajectory. Consequently, given the instrument’s relatively large FOV, the measured $\Delta $E vs. E’ distributions are significantly broader than the nominal tracks expected for normal incident particles.

To improve the energy resolution and the resulting selection efficiency, the recorded signals can be corrected for the proportionality between the deposited energy and the cosine of the inclination angle $\mathit{cos}\theta $. Particle simulations were employed to compute the particle trajectories inside the FOV. Then, modified from the approach developed for the STEREO/LET detector, the desired transformation is accomplished by multiplying both $\Delta $E and E’ by the appropriate factor where the power-law index $a$ was obtained with the following iterative procedure, optimized for providing the highest ^3^He/^4^He discrimination.

**Fig. 11:**
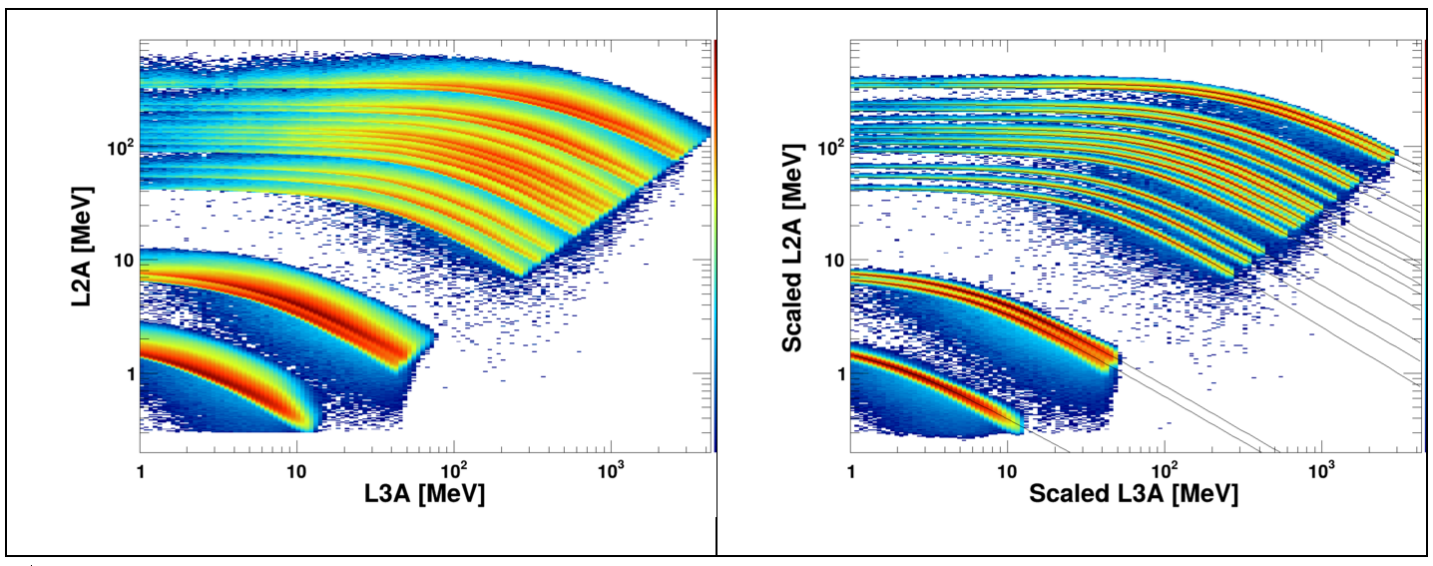
Monte Carlo simulation of the HIT response to particles incident from the A side and stopping in the L3A detectors. The left-hand panels show the actual energy losses while the right-hand panels show the same events after approximate correction for variations in incidence angle L_*θ*_, as described in the text; the double-power-law fits of the tracks are also displayed

The effect of the cosine correction is demonstrated in Fig. 11, displaying the ion tracks for Range3. The left-hand panel shows the raw pulse height, while the right-hand side of the figure displays how the events can be mapped onto well-separated element tracks based on the scaling procedure that is used onboard to approximately correct for spread due to particle trajectory inclination.

### Charge Algorithm

HIT sends down a portion of the raw (unbinned) “event” data including pulse heights in each detector which will be used to calculate Level 3 data products, including the electric charge, Z, of the measured ion species. The developed approach relies on Geant4 simulations of the instrument response (see Sect. 4.1).

## Calibration

### Electronic Calibration

The PHASIC can simulate events by injecting a controlled amount of charge into the preamp inputs for any combination of channels simultaneously using a single logic signal as a trigger and a DC voltage as a reference to set the magnitude. Each channel has its own array of charge injection capacitors, programmable from 0 to 60 picofarads in 4 picofarad increments.

The FEE board generates the test reference voltage with a 12-bit digital-to-analog converter (DAC) with a reference that is switchable between 5.0 V and 0.505 V, giving it a high and low range. The DAC and DAC range are set by the FEE FPGA which also generates the test pulse at a programmable rate and loads the PHASIC configurations by command from the flight software. The calibration sequence can be controlled directly by command, as was mostly used for ground testing, or run autonomously by the flight software during science data collection, as intended for flight. These internal pulses can be used to calibrate the response of the PHASIC to different input charges and also simulate events to fully test out the trigger conditions.

### Radioactive Source Calibration

**Fig. 12:**
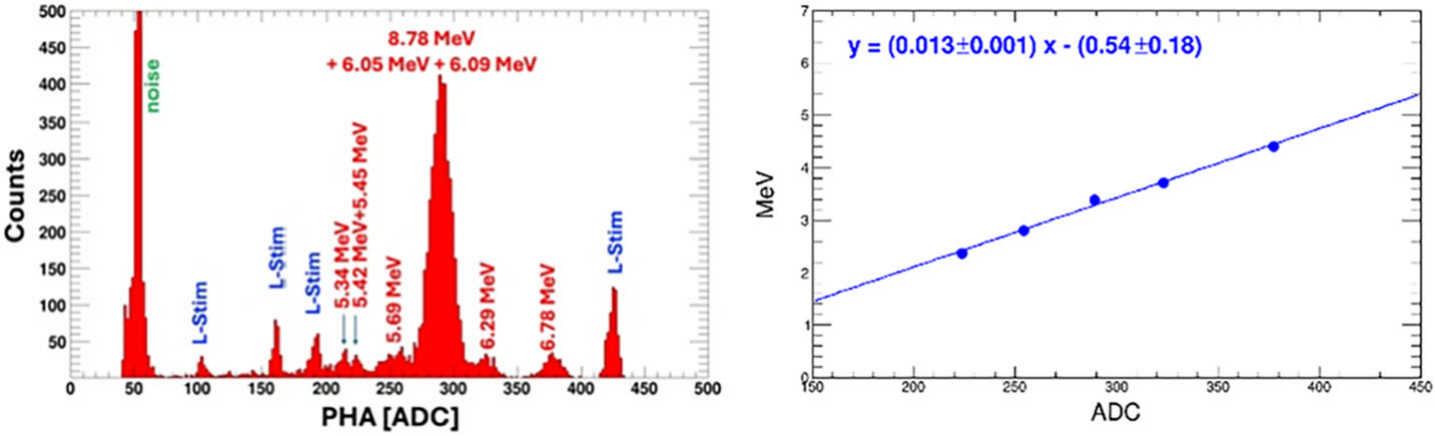
Calibration of the L1A2 detector with the ^228^Th source. Left: spectrum produced by the different $\alpha $ particles, with corresponding incident energy values; a few live-stim lines are also visible. Right: the resulting calibration curve using the distributions estimated with Geant4 simulations; the fit parameters are also reported, along with corresponding uncertainties

The HIT instrument was tested using a ^228^Th source. The decay chain of ^228^Th emits several monoenergetic helium nuclei, making ^228^Th an excellent $\alpha $-calibration source. The most dominant ones are 5.340, 5.685, 6.288, 6.778 and 8.785 MeV; the last one is energetic enough to generate a statistically significant signal in the L2 sensors when HIT is in a vacuum. Figure 12 shows the ^228^Th test results for the L1A2 detector. The left panel displays the spectrum produced by the $\alpha $ particles, with peaks identified with the aid of simulations; a few peaks associated with internal pulsers are also visible. The resulting ADC-to-MeV calibration is shown in the right panel, along with the fit results.

### Accelerator Calibration

#### Goddard Electron/Proton Accelerator

**Fig. 13:**
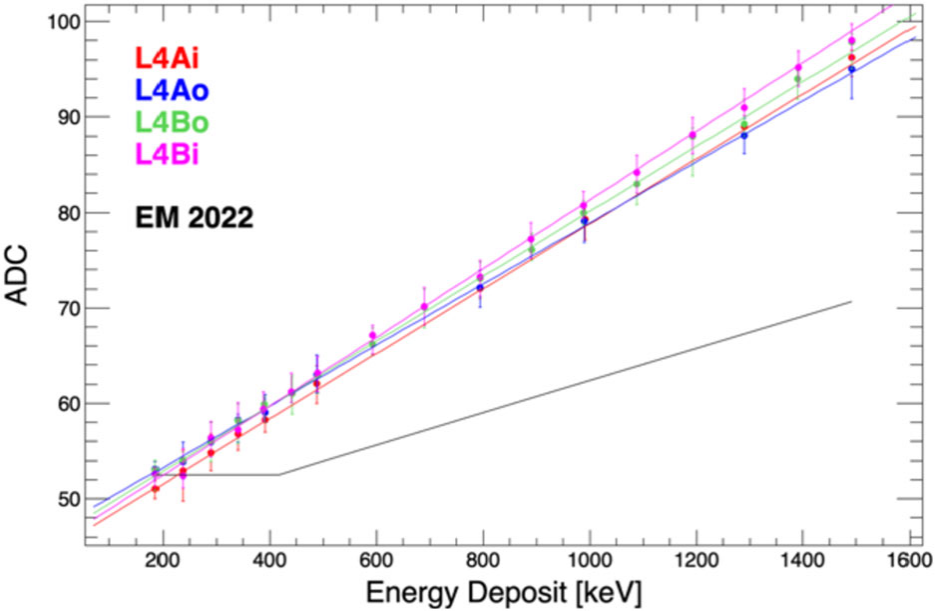
Electron calibration curves for the inner and outer segments of L4A (red) and L4B (blue). For comparison, the black line indicates a previous calibration performed in 2022 with the instrument engineering module

The first beam test was conducted at the Van de Graaff accelerator of the GSFC Radiation Effects Facility, capable of providing beams of either protons or electrons with an energy range from 100 keV to 1.7 MeV in vacuum. By using different beam energies and incident angles, we characterized the response, including the minimum detectable energy of the L1 and L4 detectors to protons and electrons, respectively. Furthermore, the electron data were used to derive an energy calibration of the L4 sensors based on the comparison with the Geant4 simulations. The best-fit curves for the inner and outer segments of the L4A (red) and L4B (blue) detectors are displayed in Fig. 13. Results are consistent within the fitting errors. An earlier calibration run on the EM is shown in black. The gain of the engineering module (EM) was a factor of two lower and the minimum measurable energy loss is roughly 400 keV, where the ADC signal becomes horizontal (the noise level). The FM calibration demonstrates an improvement by a factor >2 in terms of the minimum detectable energy. This is due to the increased gain and improved (lower noise) printed circuit board layout.

#### Brookhaven Tandem

**Fig. 14:**
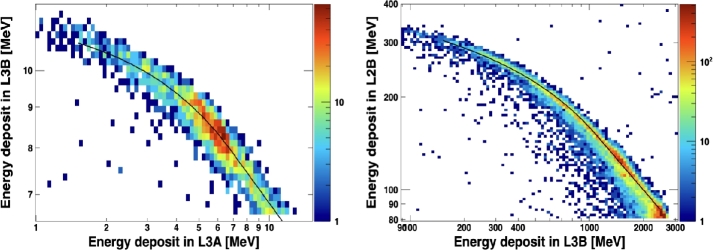
Results of the beam tests at the BNL facilities. Left: L3B vs. L3A energy distribution using a hydrogen beam at the Tandem Van der Graaf. Right: L2B vs. L3B iron energy distribution using an iron beam at the NSRL. For comparison, the black lines mark the fits of the mean values based on Monte Carlo simulations

The HIT response to relatively low-energy ions was studied at the Tandem Van de Graaff Facility at the Brookhaven National Laboratory (BNL). Specifically, we used mono-energetic H, O, and Si ion beams (see Table 7), with energies corresponding to Range4, Range3 and Range2 events, respectively. These measurements were used to determine an ADC-energy calibration for both high-gain and low-gain channels, and test dynamic threshold modes. The left panel in Fig. 14 shows the L3B vs. L3A energy distribution obtained by using the hydrogen bin. For comparison, the black line is the fit of the mean values based on Monte Carlo simulation.

**Table 7:** List of ion species and energies used at the Brookhaven National Laboratory facilities

Facility	Ion species	Energies (MeV/n)
Tandem	H	5, 15, 20
Tandem	O	6, 7.79
Tandem	Si	4.62, 6.51
NSRL	Fe	129, 142, 149, 155, 190

#### Brookhaven NSRL

We were also able to take advantage of a short run at the NASA Space Radiation Laboratory (NSRL) using high energy (∼100 MeV/n) Fe beams. Specifically, five monoenergetic beams were used (see Table 7). In order to reduce the energy of incident ions and obtain a spread of particle energies, we used a thin plastic mesh material in front of the selected instrument aperture. In addition, we employed a thin copper layer to produce lighter ions by fragmentation and spallation reactions. Results are displayed in the right panel of Fig. 14, showing the L3B vs. L3A energy distribution. For comparison, the black line indicates the fits of the mean values obtained with simulations.

### Cross-Calibration with CoDICE

**Fig. 15:**
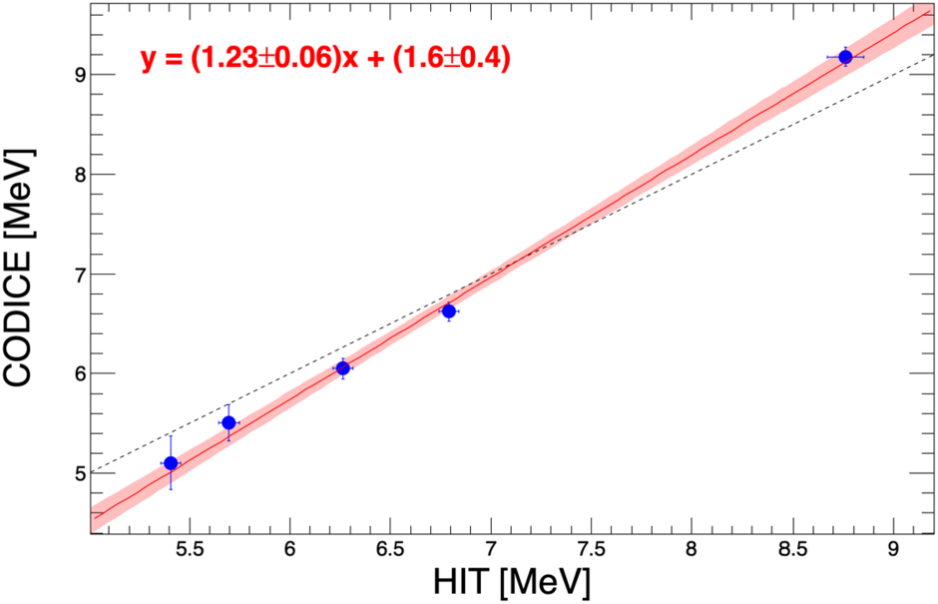
Cross-calibration for the initial particle energy of the ^228^Th alphas. Initial energies are derived using measured instrument energy losses and a GEANT model. The black dashed line what would be obtained if both instruments had perfect energy resolution

The ion observations from HIT are combined with the ions measured by CoDICE (as well as SWAPI at low energies) to generate energy spectra that span a wide energy range, key to understanding the acceleration of these ions from the thermal population seen by SWAPI, through the suprathermal energies measured by SWAPI and CoDICE, to the higher energies SEPs observed by CoDICE and HIT. HIT and CoDICE overlap in energy, but the two instruments, with very different measurement techniques and acceptance geometries needed to be cross-calibrated to remove systematic differences between the two measurements. A ^228^Th source as described in Sect. 5.2 conveniently generates multiple lines of nearly-monoenergetic alpha particles in the region of overlap. The data shown in Fig. 12 shows the energy calibration for HIT using the ^228^Th alphas and the HIT GEANT model to match the energy loss and the initial energy. However, this calibration was limited by the use of a small-area planar particle source, making it hard to assess the intensity calibration between HIT and CoDICE. Therefore, flight data is also used to improve the comparison between the two detectors based on the measurement of omnidirectional particle fluxes in interplanetary space. The intensities measured by HIT and CoDICE will also be compared to other observations at L1, for example those from ACE. This will, however, still leave some uncertainty as to the absolute intensity. Figure 15 shows the comparison between the HIT calibrated initial energy and the uncalibrated CoDICE initial energy. CoDICE still needs to finish their calibration, but this data is sufficient to match the energy scales of the two instruments to 5% or less.

## Sensor Operations

The HIT instrument has purposefully designed its operational procedures to fit the phrase “turn us on and leave us on”. To that end, HIT is not powered down for spacecraft maneuvers or activities, including thruster firings. HIT also does not require any routine commanding for nominal operations. The default mode for HIT is Science Mode in which the SSD bias voltage is on and science data is recorded at the nominal cadence.

HIT uses onboard electronic pulsers to autonomously produce “Livetime STIM” data to provide a measure of instrument livetime. The events are generated in specific detectors with several pulse height levels designed to fall within certain boxes in the event matrices and thus not interfere with science data. A separate set of autonomous pulses produce ADC-calibration STIM events which are used to monitor the stability and linearity of the pulse height analysis system. These events do not match the triggering pattern of a real particle detection and are flagged as STIM events for further analysis.

## Data Products and Algorithms

HIT has 3 primary science data products: Science Rates (Species/Energy), Summed Rates, and Sector Rates. HIT also produces I-ALiRT data products and lower-level supplementary rates. A fraction of the measured particles are also telemetered as raw “Event” data which includes the raw pulse heights in each detector in which the given particle deposited energy. The fraction of events for which raw Event data is saved is dependent on the measured particle flux at a given time.

Counts for H, He (3He and 4He), C, N, O, Ne, Na, Mg, Al, Si, S, Ar, Ca, Fe, and Ni are organized by species and energy bin for the ion apertures (A1-4 and B1-4). Particles which pass certain onboard processing cuts are sorted into matrices according to their penetration range in the instrument and by $\Delta $E by E’ measurements. Each matrix represents a penetration range – into the L2 detectors (RNG2 or L1L2), into one L3 detector (RNG3 or L2L3), or into two L3 detectors and possibly beyond (RNG4, PEN, or L3AL3B). There are also HIT ranges which include particles that pass through the I-ALiRT apertures and the L4 detector called: RNG2I (L1L4L2), RNG3I (L1L4L2L3) and RNG4I (L1L4L2L3L3). Within each penetration range, the associated matrix covers a $\Delta $E by E’ space that spans from particles with Z=1 (H) to Z ∼ 30. The Science Rates have a one-minute cadence.

The eight ion apertures (A1-4 and B1-4) and the spinning of the spacecraft are used to separate the 4$\pi $ sky into 120 sectors with eight 22.5° bins perpendicular to the spin axis (declination) and fifteen 24° bins in the spin direction (inclination). Due to the relatively small geometry factor of each sector compared to the full instrument, and corresponding reduction in counting statistics, the sector rates are accumulated for 10 minutes and are then transmitted over the next 10 minutes. These rates are divided into 10 different species/energy bins as shown in Table 8.

**Table 8:** Species and energies for the ten sectored rates

Species	H	H	H	^4^He	^4^He	CNO	CNO	NeMgSi	NeMgSi	Fe
Energy (MeV/n)	1.8-3.6	4-6	6-10	4-6	6-12	4-6	6-12	4-6	6-12	4-12

**Fig. 16:**
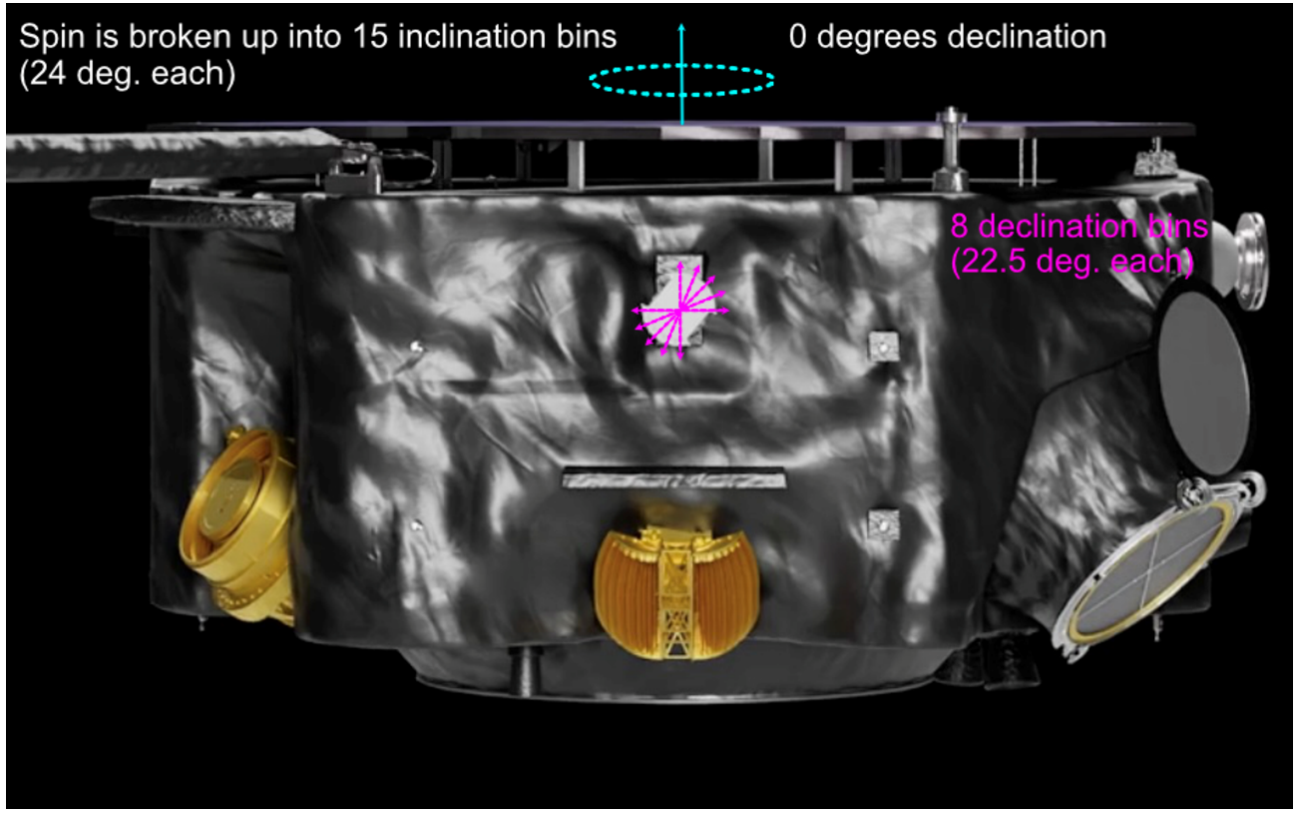
Diagram of HIT sectors. The eight declination sectors are instantaneously two fans (between the pink arrows) that sweep out the full sky as the spacecraft spins

0° in declination is defined as the spin axis in the sunward direction (several degrees away from true sunward) and the eight 22.5° bins cover the entire 180° of declination. The one-per-second time and status message from the spacecraft reports the spin phase at the start of that second. 0° in inclination is the zero of the spacecraft spin-phase. The HIT sectors are shown schematically in Fig. 16.

### Data Levels and Processing

**Fig. 17:**
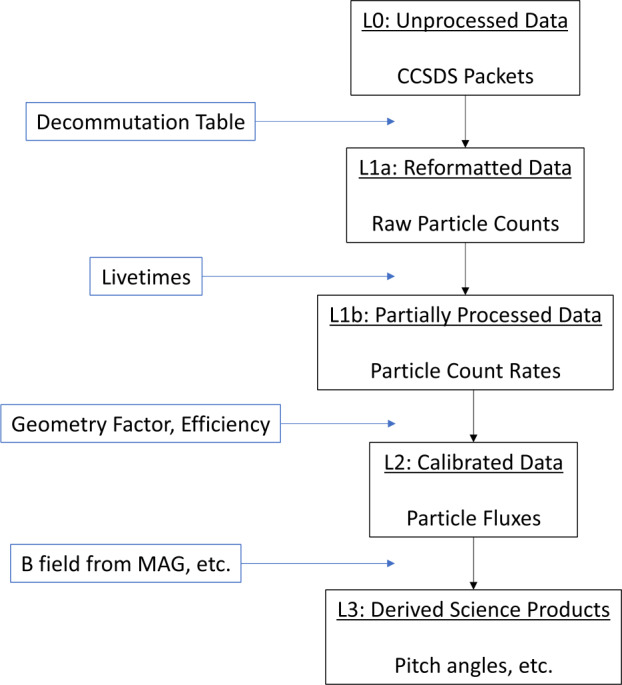
HIT data flowchart

The processing of HIT data from the raw Level 0 data transmitted by the spacecraft to partially processed (Level 1), calibrated (Level 2), and derived science (Level 3) data products are described below and in the data processing flow diagram shown in Fig. 17.

#### Level 0

Level 0 data are the original data from the instrument transmitted in the form of CCSDS files. Level 0 data are not human readable and must be de-commutated for further processing

#### Level 1A

Level 1A data come in the form of raw particle counts per integration time. These include Science Rates (species/energy binned rates), Sectored Rates, I-ALiRT Rates and a variety of supplemental rates (e.g., detector singles rates, trigger rates, priority buffer rates, etc.). Level 1A data are produced by decommutating the Level 0 CCSDS files into Level 1A CDF files. Level 1A data are classified as Reformatted Data with no further processing.

The uncertainties for the Level 1A data are calculated as asymmetric Poisson uncertainties as prescribed in Gehrels (1986). Specifically, HIT uses standard 1$\sigma $ confidence limits. For upper limits, we use Eq. 10 from Gehrels (1986) with S = 1 such that the upper uncertainty is:

$$ \delta _{u} = \sqrt{n+1} +1, $$

where n is the number of counts in a particular Level 1A variable/integration time. Equation 13 from Gehrels (1986) is used to calculate the lower uncertainty as:

$$ \delta _{l} = \sqrt{n} $$

#### Level 1B

Level 1B data are classified as Partially Processed Data as the processing converts the raw counts in Level 1A to count rates by factoring in the instrument livetime. The livetime is derived from the livetime counter based on the number of Livetime STIM pulses measured and recorded during a given integration time. The fractional livetime is then calculated for each integration time as:

$$ \mathit{Livetime}= \frac{\mathit{Livetime}\ \mathit{counter}}{270} $$

And thus the conversion from raw counts in Level 1A to count rates in Level 1B is performed as:

$$ \mathit{Count}\ \mathit{Rates}= \frac{\mathit{Raw}\ \mathit{Counts}}{\mathit{Livetime}} $$

In Level 1B, specified Science Rates (species/energy) are also summed to form the Summed Rates products. Summed Rates are described in greater detail below. The Level 1B uncertainties are calculated by dividing both the upper and lower uncertainty values calculated in Level 1A by the livetime.

#### Level 2

Level 2 HIT data are calibrated data in physical intensity units (specifically counts s^−1^ sr^−1^ cm^−2^ (MeV/n)^−1^). The conversion of the Level 1B livetime-corrected count rate data to physical units requires the species- and energy-dependent geometry factors, energy bin widths, and detection efficiencies. The intensity calculation is performed for the ion intensity in the i^th^ energy bin for a given species as:

$$ j_{i} \left ( N, X \right ) = \frac{1}{\Delta t\Delta E_{i}} \frac{\sum _{j=2}^{4} q_{ij} \left ( N, X \right ) - b_{ij} \left ( N,X \right )}{\sum _{j=2}^{4} \sigma _{ij} \left ( N, X \right ) \lambda _{ij} \left ( N,X \right )} $$

With the following symbol definitions:

$N$: ion species

$X$: look direction

$i$: energy bin

$j$: instrument range

$q$: particle counts from telemetry

$\Delta $t: integration time in units of s (60 in the case of Science Rates, 600 in the case of Sectored rates)

$\Delta $E: energy bin width

$\sigma $: geometry factor

$\lambda $: detection efficiency

b: background counts

The geometry factor and detection efficiencies are also dependent on the dynamic threshold state of the instrument at the time of measurement. These factors have been calculated via detailed instrument Monte-Carlo simulation.

#### Level 3

The calibrated HIT Level 2 data are further processed to calculate the Level 3 derived science data products. The Level 3 data produced by HIT include pitch angle distributions derived from the HIT Level 2 Sector Rates and the IMAP/MAG Level 1D data, calculations of the element using raw Pulse Height Analysis event data (algorithm described above), and science-quality electron data derived from the I-ALiRT products.

The HIT pitch angle data will combine the Level 2 Sectored Rates with the IMAP/MAG Level 1D magnetic field vector in the despun instrument frame to produce species/energy-dependent flux data in physical units with relation to the magnetic field vector. A 2D “skymap” will be produced based on the input Sectored Rates and magnetic field vector with output bins that are 22.5° in declination and 24° in inclination.

HIT utilizes the raw PHA “event” data in Level 0 to calculate the species of each particle for which event data is available as described above. This will allow a more detailed examination of the composition of a given event beyond what is possible with the Science Rates which are binned in species according to the onboard matrices.

Finally, a set of science-quality electron data products are available from the Level 3 data. These products utilize the 6 HIT I-ALiRT electron products (3 from the A-side and 3 from the B-side). Producing science-quality electron products requires a detailed examination of the ion contamination in those channels and a method of reliably removing that background.

### I-ALiRT Rates

The I-ALiRT data are produced from a combination of single-parameter measurements from the L4 (I-ALiRT) detectors and the L4 and L3 detectors to produce $\Delta $E by E’ measurements. There are 6 I-ALiRT electron products corresponding to low-, medium-, and high-energy electrons separated by A-side and B-side. The low- and medium-energy electron data are single-parameter L4 measurements and the high-energy electron data are events that interact in L4 and L3. The I-ALiRT data also includes 3 proton products including an omnidirectional medium energy product produced by summing the 12–15 MeV and 15–70 MeV proton energy bins from the standard Science Rates and two high-energy proton channels (one for the A-side and one for the B-side) derived from energy deposit in the L4 and L3 detectors. HIT I-ALiRT data also includes two ^4^He products derived from the standard science rates: a low-energy (6–8 MeV/n) omnidirectional ^4^He product and a high-energy (15–70 MeV/n) omnidirectional ^4^He product.
